# Structural Basis for PPARα Activation by 1H-pyrazolo-[3,4-b]pyridine Derivatives

**DOI:** 10.1038/s41598-020-64527-x

**Published:** 2020-05-06

**Authors:** Takuya Yoshida, Hiroya Oki, Michihiro Doi, Syohei Fukuda, Tomohiro Yuzuriha, Ryotaro Tabata, Kenji Ishimoto, Kazuki Kawahara, Tadayasu Ohkubo, Hiroyuki Miyachi, Takefumi Doi, Keisuke Tachibana

**Affiliations:** 1 Graduate School of Pharmaceutical Sciences, Osaka University, 1-6 Yamadaoka, Suita, Osaka, 565-0871 Japan; 2 Present Address: Faculty of Pharmaceutical Sciences, Setsunan University, 45-1 Nagaotoge-cho, Hirakata, Osaka, 573-0101 Japan; 3 Drug Discover Initiative, University of Tokyo, 7-3-1 Hongo, Bynkyo, Tokyo, 113-0033 Japan

**Keywords:** Structural biology, Drug discovery

## Abstract

Small-molecule agonism of peroxisome proliferator-activated receptor α (PPARα), a ligand-activated transcriptional factor involved in regulating fatty acid metabolism, is an important approach for treating dyslipidemia. Here, we determined the structures of the ligand-binding domain (LBD) of PPARα in complex with 1H-pyrazolo[3,4-b]pyridine-4-carboxylic acid derivatives, which were recently identified as PPARα-selective activators with markedly different structures from those of the well-known PPARα agonists fibrates. The crystal structures of the complexes showed that they form a canonical hydrogen-bond network involving helix 12 in the LBD, which is thought to be essential for PPARα activation, as also observed for fibrates. However, the phenyl side chain of the compounds occupies a small cavity between Ile272 and Ile354, which is rarely accessed by fibrates. This unique feature may be essential for subtype selectivity and combine with the well-characterized binding mode of fibrates to improve activity. These findings demonstrate the advantage of using 1H-pyrazolo-[3,4-b]pyridine as a skeleton of PPARα agonists and provide insight into the design of molecules for treating dyslipidemia.

## Introduction

Peroxisome proliferator-activated receptors (PPARs) are ligand-dependent transcriptional factors that belong to the NR1C class of the nuclear receptor superfamily^1^. PPARs heterodimerize with retinoid X receptor (NR2B)^2^ and recognize peroxisome proliferator-responsive elements in the promotor region of target genes involved in lipid and glucose metabolism, adipogenesis, and inflammation^3^. They share structural elements, with an N-terminal A/B domain containing a ligand-independent activation function 1 (AF-1), DNA-binding domain (DBD), hinge region, and C-terminal ligand-binding domain (LBD) harboring the ligand-dependent activation function 2 (AF-2) and interface for heterodimerization^4^. The LBD of PPAR comprises a three-layered α-helical sandwich fold, which is composed of twelve α helices and a three-stranded antiparallel β sheet, with a Y-shaped ligand binding pocket (LBP)^5^. Binding of a PPAR agonist to the LBP induces a conformational change in helix 12 (H12, also known as AF-2 helix) and organizes the AF-2 surface, which is composed of helices 3, 4, 5, and 12 to accommodate a transcriptional coactivator, such as peroxisome proliferator-activated receptor gamma coactivator 1 alpha (PGC1α)^6,7^.

Among the three known subtypes (α, β/δ, and γ), PPARα (NR1C1) was the first identified and is mainly expressed in the liver, as well as in the heart, muscle, and kidney, where fatty acid β-oxidation is promoted^8^. Activation of PPARα results in a substantial reduction in serum triglycerides, increase in HDL cholesterol, and improvement in insulin sensitivity^9^. Therefore, PPARα has been recognized as a relevant drug target for metabolic syndrome, type 2 diabetes, and coronary atherosclerosis. Furthermore, recent studies demonstrated that PPARα agonists are drug candidates for nonalcoholic fatty liver disease (NAFLD) and nonalcoholic steatohepatitis (NASH) development^10–13^, and several fibrates as representative synthetic PPARα agonists have been indeed widely used to treat dyslipidemia including hypercholesterolemia and hypertriglyceridemia^14^.

**Figure 1:**
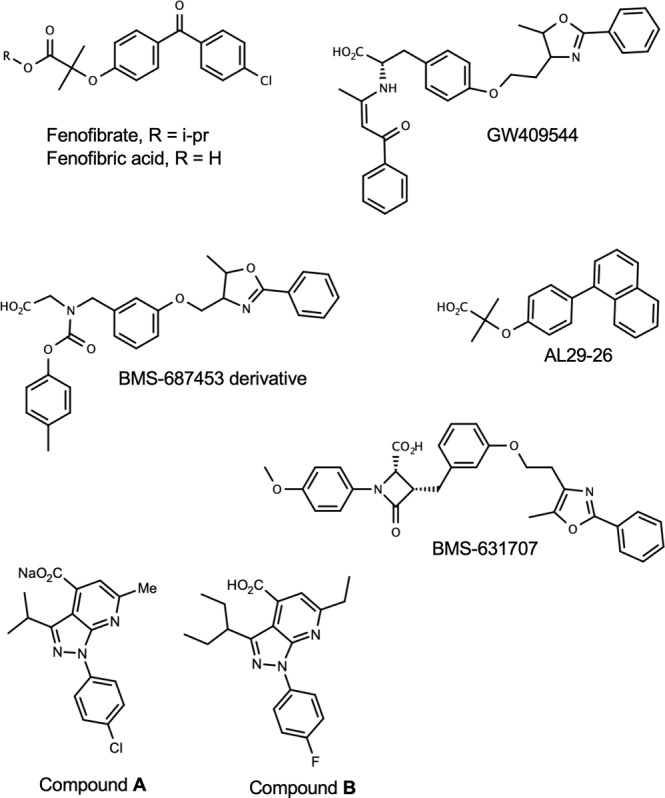
Chemical structures of PPARα agonist. Chemical structures were drawn using MarvinSketch 19.10 (ChemAxon, https://chemaxon.com/products/marvin).

Fibrates have a common structural feature containing a carboxylic head and hydrophobic tail linked by one or more aromatic rings as found in fenofibrate (Fig. 1)^15^. The structural basis of the agonistic behavior of fibrates of PPARα has been established. The carboxylic group forms a hydrogen bond, stabilizing the conformation of H12 to recruit the transcriptional coactivator, and the hydrophobic tail is important for the affinity to LBP^16^. Although fibrate-class compounds have been established as anti-hyperlipidemic agents, their relatively low affinity and subtype selectivity require improvement. Thus, studies are being conducted to develop novel PPARα-selective agonist.

We recently developed a novel screening system for PPARα agonists and discovered a new structural class these agonists, 1H-pyrazolo[3,4-b]pyridine-4-carboxylic acid derivatives (hereafter referred to as 1H-pyrazolo-[3,4-b]pyridine derivatives), which strongly activate PPARα^17^. The basic skeleton of these compounds, 1H-pyrazolo-[3,4-b]pyridine, is found in several bioactive compounds, e.g. antagonist of adenosine receptor^18^ and kinase inhibitor^19^, but greatly differ from those of known PPARα agonist. These novel ligands achieved the same level of transcriptional activation at lower concentrations than fenofibrate in a GAL4 chimera reporter assay. Furthermore, they could be expected to exhibit more potent hypolipidemic effects *in vivo* with reduced toxicities. Thus, 1H-pyrazolo-[3,4-b]pyridine derivatives could be promising lead compounds for dyslipidemia owing to their low associated risk^17^. Most recently, it was shown that the structure-activity-relationships (SAR) of 1H-pyrazolo-[3,4-b]pyridine derivatives were somewhat different from those of fibrates^20^. However, the binding mode and structural basis of activity of 1H-pyrazolo-[3,4-b]pyridine derivatives, which are important for improving their efficacy, remains unknown.

In this study, we determined the three-dimensional structures of PPARα-LBD in complex with 1H-pyrazolo-[3,4-b]pyridine derivatives. These structures showed that the carboxylic group of our compounds interacts with H12 in a similar manner as known fibrates and support the well-known mechanism of agonism via AF-2. We also found that the phenyl side chain of the compounds utilizes an overlooked cavity in the LBP. Our results provide new direction for the design of highly selective and effective agonists for PPARα.

## Results and Discussion

### 1H-pyrazolo-[3,4-b]pyridine derivatives activate PPARα selectively

**Figure 2:**
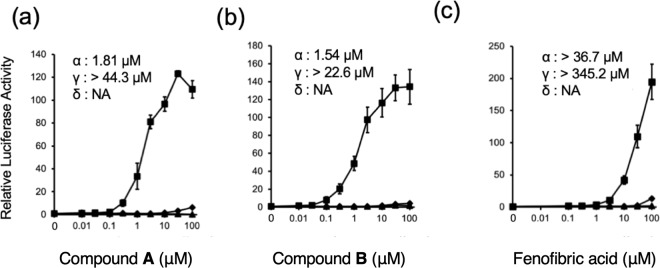
Transcriptional activation of hPPAR subtypes in GAL4 chimeric receptor assay by compounds: PPARα (square), PPARγ (circle), and PPARβ/δ (triangle). Cells were treated with various concentrations of compound **A** (a), compound **B** (b), or fenofibric acid (c). Values are expressed as fold-induction of the vehicle set as 1. Estimated EC_50_ values for subtypes are shown on each plot. For all graphs, error bars indicate the mean ± SE of three or four independent measurements.

The activation of PPARs by two 1H-pyrazolo-[3,4-b]pyridine derivatives, **A** and **B** (Fig. 1), was examined in a chimera reporter assay, where the LBD of PPARα, β/δ, or γ is fused to the GAL4 DNA-binding domain and a luciferase gene is under regulation of the GAL4 binding element (Fig. 2). Both 10 μM of compound **A** and 3 μM of compound **B** enhanced the transactivation function of PPARα by 100-fold, which was comparable to that observed for approximately 50 μM of fenofibric acid, the bioactive form of fenofibrate. We also confirmed that our compounds can activate full-length human PPARα to upregulate the promoter activity of the human solute carrier family 25, member 20 gene (SLC25A20) which is a known PPARα target gene^21^ (Supplementary Fig. S1).

### Overall structure of PPARα-LBD complexes

To gain insight into the binding mode of 1H-pyrazolo-[3,4-b]pyridine derivatives, we determined the cocrystal structures of PPARα-LBD in complex with compounds **A** and **B** at 1.95 and 1.98 Å resolutions, respectively, in the presence of PGC1α-derived peptide. These structures belong to the same space group, *P*2_1_2_1_2_1_, and have similar cell dimensions. Data collection and refinement statistics are shown in Table 1. Each asymmetric unit contained one PPARα-LBD molecule. The structures showed a typical nuclear receptor fold where H12 of LBD has an active conformation suitable for interacting with the agonist and forming a binding surface for the PGC1α peptide. Residues 200–204 of PPARα-LBD/PGC1α-peptide/**A** complex and 200–204, 234, 235, and 257–263 of PPARα-LBD/PGC1α-peptide/**B** complex could not be modeled because of the lack of electron densities. The overall structures of both complexes are similar to each other with a root-mean-square deviation of 0.28 Å for 226 Cα atoms of the LBDs.

**Table 1:** Data collection and structure refinement statistics.

	PPARα-LBD / Compound A/ PGC1α	PPARα-LBD / Compound B/ PGC1α
**Data collection**		
Wavelength(Å)	1.0000	1.0000
Space group	*P*2_1_2_1_2_1_	*P*2_1_2_1_2_1_
**Unit cell dimensions**		
*a,b,c* (Å)	45.06, 60.68, 100.34	45.55, 61.20, 103.37
α, β, γ (°)	90	90
Resolution range (Å)^*a*^	45.06–1.95 (2.00–1.95)	45.55–2.00 (2.05–2.00)
No. of unique reflections	20,564 (1417)	20,224 (1454)
Completeness (%)	99.3 (99.3)	100.0 (100.0)
Redundancy	12.7 (13.4)	12.4 (12.7)
*I/σ(Ι)*	19.2 (2.3)	12.6 (2.3)
*R*_*merge*_^*b*^	0.073 (1.179)	0.107 (1.196)
CC_1/2_^*c*^	0.999 (0.756)	0.998 (0.804)
**Refinement**		
Resolution range (Å)	29.04–1.95	30.03–2.00
*R*_*work*_*/R*_*free*_	0.1925/0.2309	0.2180/0.2520
RMSD from ideal values		
Bond length (Å)	0.005	0.004
Bond angles (°)	0.98	0.93
Overall B-factor (Å^2^)	40.6	44.5
Ramachandran plot statistics (%)		
Most favored	98.2	99.2
Allowed	1.9	0.8
Disallowed	0	0

^a^Values in parentheses are for the highest resolution shell.

^b^*R*_merge_ = ∑_*hkl*_∑_*i*_ | *I*_*i*_(*hkl*) - <*I*(*hkl*)>|/∑_*hkl*_∑_*i*_*I*_*i*_(*hkl*), where *I*_*i*_(*hkl*) is the intensity of the *i*-th measurement of an equivalent reflection and <*I*(*hkl*)> is the average intensity for multiply recorded reflections.

^c^CC_1/2_ is the Pearson correlation coefficient calculated between two random half data sets.

### Structural basis of agonism of 1H-pyrazolo-[3,4-b]pyridine derivatives

**Figure 3:**
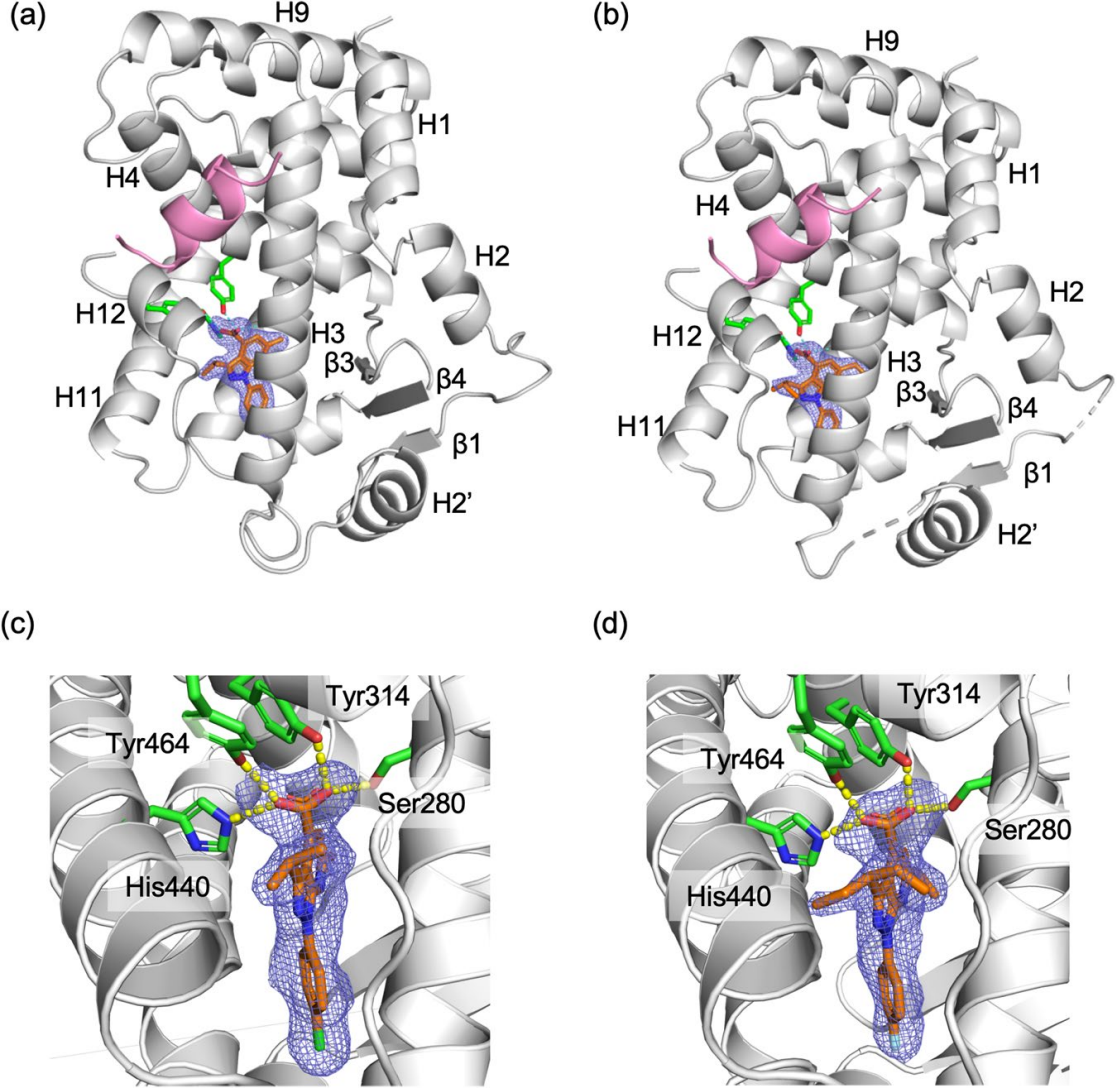
Crystal structures of hPPARα LBD bound to 1H-pyrazolo-[3,4-b]pyridine derivatives. (a and b) Overall structures of PPARα LBD in complex with compounds **A** and **B**, respectively. The PPARα LBDs are drawn as a cartoon model (light gray) with PCG1α-derived peptide (pink). Compounds are shown as a stick model (orange) with the *mF*_*o*_*-DF*_*c*_ omit electron density map (1.5σ). The residues interacting with the carboxylic acid moiety are presented as a stick model (green) with hydrogen bonds drawn by a sashed line. (c and d) Close-up views of interactions between PPARα LBD and compound **A** and **B**, respectively. These figures have been created with PyMol 2.3 (Schrödinger LLC, https://pymol.org).

Clear electron densities in the ligand omit maps revealed that both compounds, **A** and **B**, occupied same site in the LBD with the same orientation (Fig. 3). Although compound **A** was prepared as a sodium salt, it was found to exist in an acid form, which was also observed for compound **B**. Inside the LBP surrounded by H3, H5, H7, H11, H12, and β3, the compounds were close to the side of H12, which was located on the opposite side of β3. One of the two oxygen atoms of the carboxylic group of the compounds forms a bifurcated hydrogen bond with the Oγ atom of Ser280 (H11) and Oη atom of Tyr314 (H5). The other oxygen atom makes a bifurcated hydrogen bond with the Nε atom of His440 (H11) and Oη atom of Tyr464 (H12). Among them, the interaction with Tyr464 on H12 appears to be important for stabilizing the conformation of H12 in the active form, where the carboxylic group of Glu462 of H12 forms hydrogen bonds to Oγ of Ser142, and backbone amide moieties of Leu143 and Leu144 in the PGC1α-peptide. This hydrogen-bond network has been commonly observed in known structures of PPARα-LBD/agonist complex and contributes to the structural stability of PPARα-LBD^22^. Therefore, it is considered that our compounds act in the same way as other PPARα agonists including fibrates.

**Figure 4:**
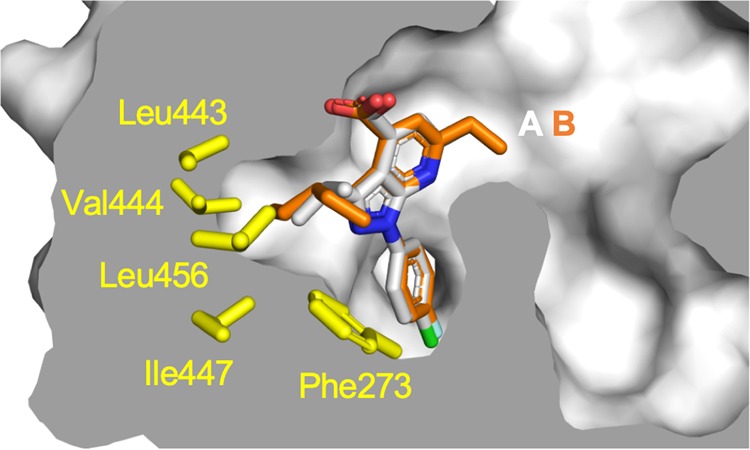
Superpositions of compounds **A** and **B** bound to PPARα LBD. Compounds **A** and **B** are represented by white and orange stick models, respectively. A cross-section of the binding pocket is depicted with a gray surface. The sidechain of interacting amino acid residues of a hydrophobic cavity around the loop connecting helix 11 and 12 are depicted as yellow stick models. This figure has been created with PyMol 2.3 (Schrödinger LLC, https://pymol.org).

On the pyrazole ring of the compounds, branched aliphatic substituents formed contacts with the loop region connecting H11 and H12, where a small hydrophobic cavity is formed by Phe273 (H3), Leu443, Val444, Ile447 (H11), and Leu456 (H11-H12 loop) (Fig. 4). Among them, Phe273 was reported to be important for the stability of active conformation of H12 from molecular dynamics simulation^22^. Compared to the isopropyl group in **A**, the bulkier 3-pentyl group in **B** seems better suited for filling the cavity. Considering that compound **B** is slightly more active than **A** (Fig. 2), these hydrophobic groups in this site may contribute to stabilizing the active conformation of H12. Indeed, it has been shown that smaller substituents such as a methyl or cyclopropyl group at this position decreased the activity^20^. In the previously reported structure of PPARα-LBD complexed with BMS-631707 (PDB ID: 2REW), a methoxy benzene ring was buried in the cavity. In the case of the complex with a PPAR pan-agonist, L29–26, a larger naphthyl ring was inserted and distorted the structure of the H11-H12 loop (PDB ID: 5HYK)^23^. The observed diversity and tolerance of interacting groups indicate the flexibility of this loop. Thus, further optimizations of the aliphatic moieties may improve the activity of 1H-pyrazolo-[3,4-b]pyridine derivatives.

### PPARα-specific binding by characteristic interactions of p-halophenyl substituent

**Figure 5:**
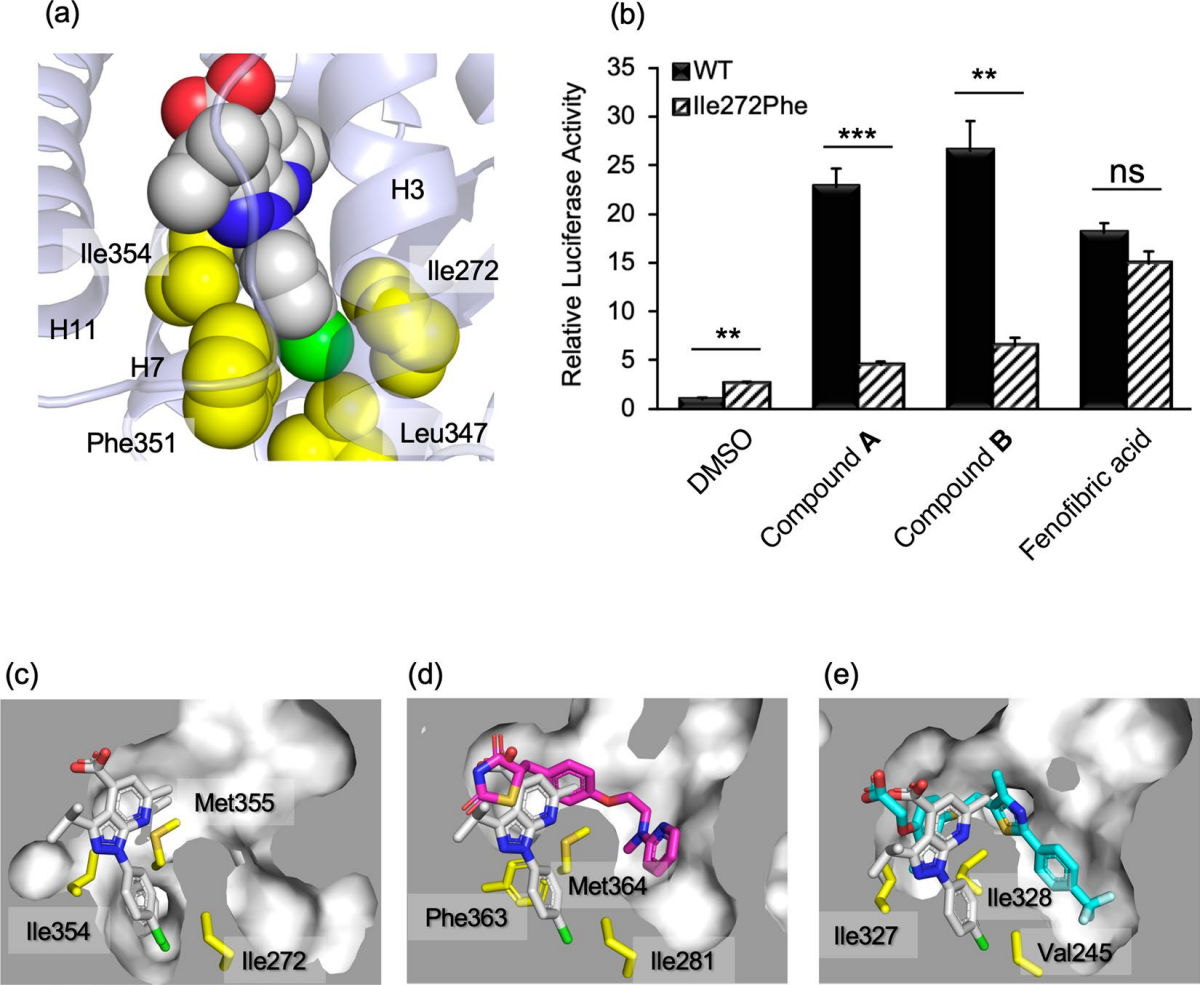
Interactions of the *p*-halophenyl substituent of the 1H-pyrazolo-[3,4-b]pyridine derivative to the hydrophobic cleft between helices 3 and 7. (**a**) Compound **A** is represented by a space-filling model (C: white, N: blue, O: red, and Cl: green). The sidechains of interacting amino acid residues are depicted as yellow space-filling models. (**b**) Transactivation of WT and Ile272Phe mutant of hPPARα LBD by compounds. Cells transfected with the reporter plasmid were treated with 1 μM of compounds **A**, **B**, 10 μM of fenofibric acid, or 0.1% dimethyl sulfoxide (vehicle). The free acid form of compound **A** was used in this assay. Values are expressed as the fold-induction compared to the vehicle, which was set to 1. For all graphs, error bars indicate the mean ± SE of three independent measurements. Significant differences between the values of WT and Ile272Phe were determined using Student’s *t*-test (*P < 0.05, **P < 0.01, and ***P < 0.001). (**c**) Close-up views of interactions between PPARα LBD and compound **A** (white), (**d**) PPARγ LBD and rosiglitazone (magenta), and (**e**) PPARβ/δ LBD and GW501516 (cyan). Cross-sections of the binding pockets are depicted with a gray surface. The binding position of compound **A** is shown in (d) and (e). These figures have been created with PyMol 2.3 (Schrödinger LLC, https://pymol.org).

The other substituent on the pyrazole ring, a *p*-halophenyl (*p*-chlorophenyl for **A** and *p*-fluorophenyl for **B**) group, was accommodated in a narrow flat cavity surrounded by the side chains of Ile272 (H3), Leu347 (H6), Phe351 (H7), and Ile354 (H7) (Fig. 5a). Notably, two side-chain methyl groups, γ2 of Ile272 and δ1 of Ile354, sandwich the halophenyl ring, suggesting that CH-π interactions stabilize the binding and contribute to the high affinity of our compounds for PPARα-LBD. To confirm the contribution of these interactions, the mutation Ile272Phe was introduced. A transactivation assay (Fig. 5b) demonstrated that Ile272Phe mutation nearly abolished transactivation by 1H-pyrazolo-[3,4-b]pyridine derivatives, while fenofibric acid showed comparable activity between WT and Ile272Phe. These results indicate that shape complementarity in this cavity is essential for the activity of 1H-pyrazolo-[3,4-b]pyridine derivatives. The SAR study, in which substitution at this position with an *m*-halophenyl ring was not allowed^20^, supports the importance of the steric contribution at this site.

In addition, α-subtype selectivity for PPAR of the compounds were attributed to the interactions in this cavity (Fig. 5c–e). In the PPARγ-LBD/rosiglitazone complex (PDB ID: 5YCP)^24^, the cavity is filled with the bulky sidechain of Phe363, which corresponds to Ile354 (H7) of PPARα (Fig. 5d). Thus, to accommodate *p*-halophenyl group in the LBP of PPARγ, this residue should move. In the complex of PPARγ and a PPARα/γ dual agonist, GW409544, it has been observed that the cavity is filled by a phenyl moiety of the compound and Phe363 is flipped out. However, trace activities of 1H-pyrazolo-[3,4-b]pyridine derivatives toward PPARγ (Fig. 2) indicate that such re-organization of the structure can occur, but the energy barrier is higher than for the structure of 1H-pyrazolo-[3,4-b]pyridine derivatives. In contrast, the *p*-halophenyl group may interfere with the branched sidechain of Ile328 in PPARβ/δ-LBD/GW501516, which corresponds to Met355 (H7) of PPARα (Fig. 5e). The complete loss of the activity of the compounds against PPARβ/δ (Fig. 2) suggests that this steric hindrance cannot be overcome by the induced-fit mechanism.

### Structural comparison to known PPARα ligands

**Figure 6:**
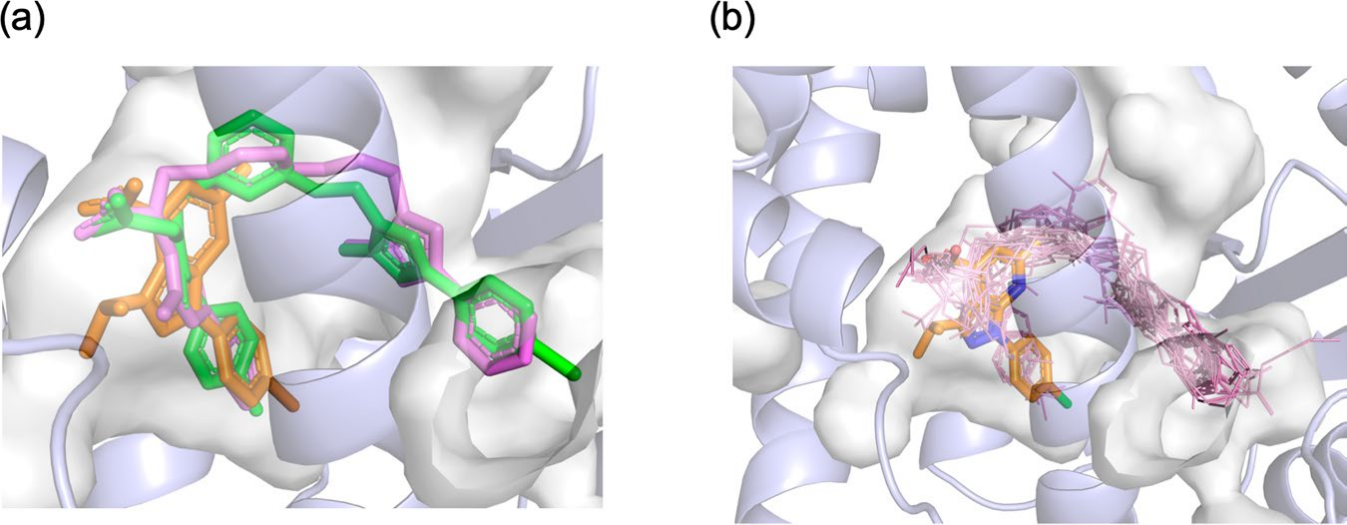
Superpositions of PPARα ligands from reported complex structures. (**a**) Compound **A** (orange) is overlaid on GW409544 (magenta) and BMS-687453 derivative (green). (b) Ligand structures in PDB (1I7G, 1K7L, 1KKQ, 2NPA, 2P54, 2ZNN, 3ET1, 3FEI, 3G8I, 3KDT, 3KDU, 3SP6, 3VI8, 4BCR, 4CI4, and 5AZT) are shown in lines overlaid onto compound **A** represented by orange stick model. The binding pocket is depicted with a gray transparent surface. These figures have been created with PyMol 2.3 (Schrödinger LLC, https://pymol.org).

Interestingly, within the reported structures of PPARα-LBD/agonist complexes, there are a few cases in which the cavity between Ile272 and Ile354 is filled by ligands. In the complex with a BMS-687453 derivative (compound 12, PDB ID: 3KDU)^25^, a *p*-methylphenyl group was located in a similar position (Fig. 6a). In both of our compounds and the BMS-687453 derivative, the carboxylic acid group participating the hydrogen bonding network involving H12 and the phenyl moiety are connected through four atoms, which are in the same plane as the carbamoyl moiety of the BMS-687453 derivative or 1H-pyrazolo-[3,4-b]pyridine rings of our structures. These observations suggest that the structures applicable to fit this pocket are limited to a relatively narrow chemical space. Notably, it was reported that the 4-methylphenyl group of the BMS-687453 derivative makes a lower contribution to its affinity to LBD. While SAR remain to be investigated, a rigid ring structure of 1H-pyrazolo-[3,4-b]pyridine may be advantageous over the carbamoyl moiety for locating the interacting groups in a more favorable manner with a low entropic penalty.

In contrast to substituents on the pyrazole moiety, those on the pyridine ring (methyl for **A** and ethyl for **B**) are small and do not appear to responsible for binding of the compounds. On this side of the compound, LBP opens towards the solvent and is divided by β3 into two regions toward H5 and H6, which are often referred to as the entrance and arm-II^5^, respectively. In nearly all previously reported structures of PPARα-LBD/agonist complexes, the long hydrophobic moiety of the ligand occupies the arm-II region towards H6, as shown in Fig. 6b, and would be essential for correctly positioning the carboxylic acid group nearby H12. The differences among known fibrate class compounds can be attributed to structural variations in these hydrophobic moieties. Recently, a complex structure of new potent PPARα modulator, pemafibrate, with PPARα LBD was reported^26^. In this structure, the branched side chains of pemafibrate made contact with the entrance regions in addition to the arm II region. Our ligands, however, do not rely on interactions with these regions in LBP to achieve high activity, indicating the high complementarity of the molecular shape between the 1H-pyrazolo-[3,4-b]pyridine derivatives and H12 side of LBP. Therefore, the activities of our ligands may be enhanced by introducing adequate substituents already tested in fibrates to fill the region toward H6 in the LBP.

## Conclusion

In conclusion, we demonstrated that the 1H-pyrazolo-[3,4-b]pyridine derivatives bind selectively to PPARα-LBD and activate PPARα with high efficacy because of the near-optimal arrangement of the interacting groups, carboxylic group for H12, branched aliphatic substituents for the H11-H12 loop, and halophenyl substituents for Ile272 (H3) and Ile354 (H7). Furthermore, it has been suggested that the accumulated knowledge of SAR of fibrates can be applied to modify the 1H-pyrazolo-[3,4-b]pyridine derivatives by fusing them. Our study revealed the potential of using 1H-pyrazolo-[3,4-b]pyridine as a skeleton for developing potent PPARα agonists and provides clues for their structure-based optimization to develop novel drugs for dyslipidemia.

## Methods

### Chemicals

Compounds A and B were synthesized as reported previously^20^. All other chemicals were obtained from Nacalai Tesque (Kyoto, Japan) unless otherwise indicated.

### Reporter assay

The activation of PPARs by compounds was measured by using a chimeric receptor consisting of the GAL4-DNA-binding domain and PPAR-LBD with a luciferase (LUC) reporter system, p4×UAS-tk-luc, which is a LUC reporter construct containing four copies of the GAL4 binding site followed by the thymidine kinase promoter. Assays were performed as described previously^17^. Briefly, HepG2 cells were transfected with p4×UAS-tk-luc together with the pBIND-hPPARα-LBD, pBIND-hPPARβ/δ-LBD, or pBIND-hPPARγ1-LBD expression vector (an expression plasmid for GAL4-hPPAR chimera protein) using Lipofectamine 2000 (Thermo Fisher Scientific, Waltham, MA, USA) according to the manufacturer’s instructions. At 24 h after transfection, the cells were treated with each compound. After 24 h, both firefly and Renilla luciferase activities were quantified using the Dual-Luciferase reporter assay system (Promega, Madison, WI, USA) on a luminometer. The Firefly luciferase signal was normalized to the Renilla luciferase signal.

### Protein expression and purification

The synthesized gene of human PPARα-LBD (residues 200–468) was cloned into downstream of thioredoxin and hexa-histidine tag inserted in the pET vector. *Escherichia coli* BL21(DE3) cells transformed with the plasmid were grown at 37 °C in LB medium containing 100 μg/mL ampicillin to A600 = 0.6 and were induced by adding IPTG to a final concentration of 0.1 mM. Next, the cells were grown for 12–14 h at 20 °C. Harvested cells were lysed by sonication in buffer A (20 mM Tris-HCl pH 8.0, 500 mM NaCl, 10 mM imidazole, 5% glycerol). After the supernatant was loaded onto a HisTrap FF column (GE Healthcare Life Sciences, Little Chalfont, UK), the bound protein was eluted with buffer A containing 250 mM imidazole. The obtained protein was desalted and further purified using a HiTrap Q HP column. The fraction eluted by a linear NaCl gradient was incubated with TEV protease, and then the fusion tag was removed using a HisTrap FF column. PPARα-LBD protein was finally purified by gel-filtration chromatography with a Superdex 200 (GE Healthcare) in 20 mM Tris-HCl pH 7.5, 150 mM NaCl. Protein purity was evaluated by SDS-PAGE. Protein concentration was determined by measuring the absorbance of the sample at 280 nm.

### Crystallization, data collection, and structure determination

Co-crystallization of PPARα-LBD with the synthesized peptide corresponding to residues 135–156 of human PGC1α and the 1H-pyrazolo-[3,4-b]pyridine derivative **A** or **B** by using the sitting-drop vapor-diffusion method was performed at 293 K. Because the PGC1α peptide and compound **B** have poor aqueous solubility, these components were added as solutions in dimethyl sulfoxide. Crystals were obtained from drops derived from 2 μL of protein solution mixed with an equal volume of crystallization buffer (100 mM HEPES-NaOH, pH 7.4, 19–23% PEG4000, 19–23% isopropanol or 1,2-propanediol). Diffraction data were collected at 100 K on beamline BL38B1 of SPring-8 (Hyogo, Japan), and were indexed, integrated, and scaled by using XDS^27^. All structures were solved by the molecular replacement method using the PHENIX program suite^28^ with the previously reported PPARα-LBD structure (PDB ID: 1I7G)^29^ as an initial search model. Structural refinement and the addition of water molecules were performed by using Coot^30^. The final structures were checked and validated by MolProbity^31^.

## Supplementary information

Supplementary information.

## Data Availability

Structural data have been deposited in the Protein Data Bank with the accession IDs 6KXX and 6KXY.

## References

[1] Nuclear Receptors Nomenclature C (1999). A unified nomenclature system for the nuclear receptor superfamily. Cell.

[2] Evans RM, Mangelsdorf DJ (2014). Nuclear Receptors, RXR, and the Big Bang. Cell.

[3] Gervois P, Fruchart JC, Staels B (2007). Drug Insight: mechanisms of action and therapeutic applications for agonists of peroxisome proliferator-activated receptors. Nat Clin Pract Endocrinol Metab.

[4] Bain DL, Heneghan AF, Connaghan-Jones KD, Miura MT (2007). Nuclear receptor structure: implications for function. Annu Rev Physiol.

[5] Zoete V, Grosdidier A, Michielin O (2007). Peroxisome proliferator-activated receptor structures: ligand specificity, molecular switch and interactions with regulators. Biochim Biophys Acta.

[6] Molnar F, Matilainen M, Carlberg C (2005). Structural determinants of the agonist-independent association of human peroxisome proliferator-activated receptors with coactivators. J Biol Chem.

[7] Yu S, Reddy JK (2007). Transcription coactivators for peroxisome proliferator-activated receptors. Biochim Biophys Acta.

[8] Lefebvre P, Chinetti G, Fruchart JC, Staels B (2006). Sorting out the roles of PPAR alpha in energy metabolism and vascular homeostasis. J Clin Invest.

[9] Shah A, Rader DJ, Millar JS (2010). The effect of PPAR-alpha agonism on apolipoprotein metabolism in humans. Atherosclerosis.

[10] Ban S (2011). Structure-based design, synthesis, and nonalcoholic steatohepatitis (NASH)-preventive effect of phenylpropanoic acid peroxisome proliferator-activated receptor (PPAR) alpha-selective agonists. Bioorg Med Chem.

[11] Kersten S, Stienstra R (2017). The role and regulation of the peroxisome proliferator activated receptor alpha in human liver. Biochimie.

[12] Pawlak M, Lefebvre P, Staels B (2015). Molecular mechanism of PPARalpha action and its impact on lipid metabolism, inflammation and fibrosis in non-alcoholic fatty liver disease. J Hepatol.

[13] Silva AKS, Peixoto CA (2018). Role of peroxisome proliferator-activated receptors in non-alcoholic fatty liver disease inflammation. Cell Mol Life Sci.

[14] Catapano AL (2016). 2016 ESC/EAS Guidelines for the Management of Dyslipidaemias. Eur Heart J.

[15] Giampietro L (2012). Synthesis and structure-activity relationships of fibrate-based analogues inside PPARs. Bioorg Med Chem Lett.

[16] Bernardes A (2013). Molecular mechanism of peroxisome proliferator-activated receptor alpha activation by WY14643: a new mode of ligand recognition and receptor stabilization. J Mol Biol.

[17] Tachibana K (2018). Discovery of peroxisome proliferator-activated receptor alpha (PPARalpha) activators with a ligand-screening system using a human PPARalpha-expressing cell line. J Biol Chem.

[18] Tuccinardi T (2008). Substituted pyrazolo[3,4-b]pyridines as potent A1 adenosine antagonists: synthesis, biological evaluation, and development of an A1 bovine receptor model. ChemMedChem.

[19] Wenglowsky S (2011). Pyrazolopyridine inhibitors of B-RafV600E. Part 2: structure-activity relationships. Bioorg Med Chem Lett.

[20] Miyachi H (2019). Structural development of 1H-pyrazolo-[3,4-b]pyridine-4-carboxylic acid derivatives as human peroxisome proliferator-activated receptor alpha (PPARalpha)-selective agonists. Bioorg Med Chem Lett.

[21] Tachibana K (2009). Regulation of the human SLC25A20 expression by peroxisome proliferator-activated receptor alpha in human hepatoblastoma cells. Biochem Biophys Res Commun.

[22] Michalik L (2007). Combined simulation and mutagenesis analyses reveal the involvement of key residues for peroxisome proliferator-activated receptor alpha helix 12 dynamic behavior. J Biol Chem.

[23] Capelli D (2016). Structural basis for PPAR partial or full activation revealed by a novel ligand binding mode. Sci Rep.

[24] Jang JY (2018). Structural Basis for the Enhanced Anti-Diabetic Efficacy of Lobeglitazone on PPARgamma. Sci Rep.

[25] Li J (2010). Discovery of an oxybenzylglycine based peroxisome proliferator activated receptor alpha selective agonist 2-((3-((2-(4-chlorophenyl)-5-methyloxazol-4-yl)methoxy)benzyl)(methoxycarbonyl)am ino)acetic acid (BMS-687453). J Med Chem.

[26] Kawasaki M (2020). Elucidation of Molecular Mechanism of a Selective PPARα Modulator, Pemafibrate, through Combinational Approaches of X-ray Crystallography, Thermodynamic Analysis, and First-Principle Calculations. Int J Mol Sci.

[27] Kabsch WX (2010). Acta Crystallogr D Biol Crystallogr.

[28] Adams PD (2010). PHENIX: a comprehensive Python-based system for macromolecular structure solution. Acta Crystallogr D Biol Crystallogr.

[29] Cronet P (2001). Structure of the PPARalpha and -gamma ligand binding domain in complex with AZ 242; ligand selectivity and agonist activation in the PPAR family. Structure.

[30] Emsley P, Cowtan K (2004). Coot: model-building tools for molecular graphics. Acta Crystallogr D Biol Crystallogr.

[31] Chen VB (2010). MolProbity: all-atom structure validation for macromolecular crystallography. Acta Crystallogr D Biol Crystallogr.

