# Supplementary material for: Structural Basis for PPARα Activation by 1H-pyrazolo-[3,4-b]pyridine Derivatives
Source: Sci Rep. 2020 May 6;10:7623. doi: 10.1038/s41598-020-64527-x (PMC7203124; doi:10.1038/s41598-020-64527-x)
Supplement: Supplementary file 1 — Supplementary information. [file 41598_2020_64527_MOESM1_ESM.pdf]

Structural Basis for PPARα Activation by 1H-pyrazolo-[3,4-b]pyridine Derivatives

(supplementary information)

Takuya Yoshida[1†], Hiroya Oki[1], Michihiro Doi[1], Syohei Fukuda[1], Tomohiro Yuzuriha[1,2], Ryotaro Tabata[1], Kenji Ishimoto[1], Kazuki Kawahara[1], Tadayasu Ohkubo[1], Hiroyuki Miyachi[3], Takefumi Doi[1], Keisuke Tachibana[1]

[1]Graduate School of Pharmaceutical Sciences, Osaka University, 1-6 Yamadaoka, Suita, Osaka 565-0871, Japan

[2](Current address) Faculty of Pharmaceutical Sciences, Setsunan University, 45-1 Nagaotoge-cho, Hirakata, Osaka 573-0101, Japan

[3]Drug Discover Initiative, University of Tokyo, 7-3-1 Hongo, Bynkyo, Tokyo 113-0033, Japan

† Correspondence should be addressed to: yo@phs.osaka-u.ac.jp

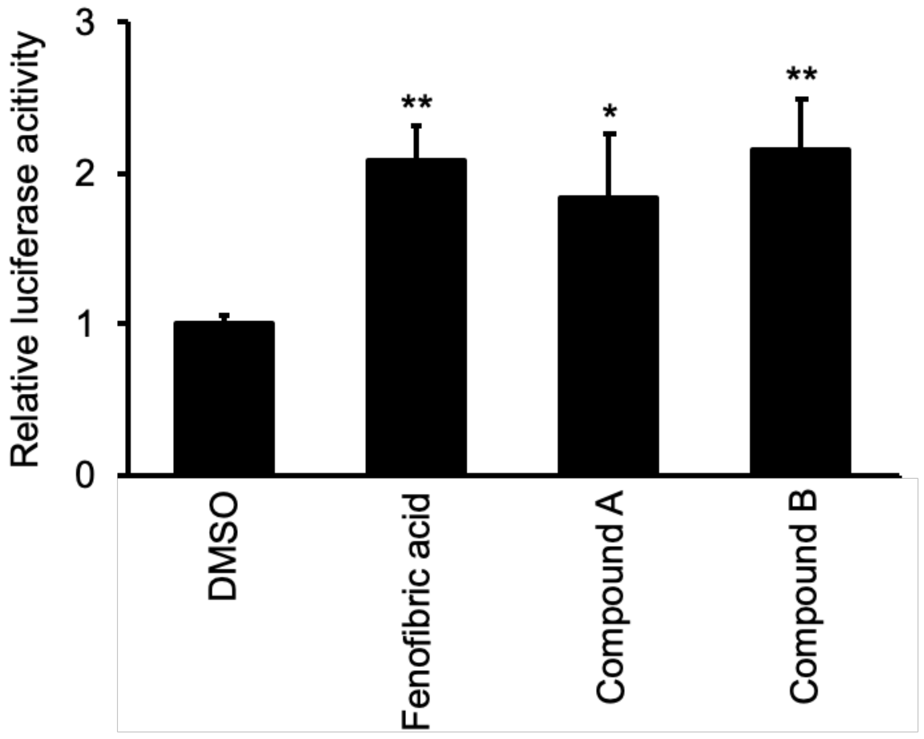

Figure S1. 1H-pyrazolo-[3,4-b]pyridine derivatives upregulate human SLC25A20 promoter activity. Solute carrier family 25, member 20 (SLC25A20) is a key molecule that transfers acylcarnitine esters in exchange for free carnitine across the mitochondrial membrane in the mitochondrial β-oxidation. PPARα directly regulates the expression of human SLC25A20 via the PPRE(Tachibana, K. et al. 2009, *Biochem Biophys Res Commun* 389, 501-505). HepG2 cells were co-transfected with the human SLC25A20 reporter plasmid (the human SLC25A20 promoter corresponding to the region -4205 to +1 bp was fused to the luciferase gene; p4205), phRL-TK, and pcDNA3-hPPARα (full-length human PPARα expression vector). Transfected cells were treated with compounds (30 μM fenofibric acid, 3 μM compound A or 3 μM compound B) or DMSO (Control) for 24 h. Luciferase activities from reporter plasmids were normalized to Renilla luciferase activities. Values are expressed as fold induction of the control set as 1. Values represent the mean ± standard deviation of triplicate samples. Reproducible results were obtained from two independent experiments. Significant differences of the values compared to the control were determined using Dunnett's test and are indicated by asterisks (* $P < 0.05$, ** $P < 0.01$). This result shows that 3 μM compounds A and B upregulate human SLC25A20 promoter activity as well as 30 μM fenofibric acid.
